# Diseases of the Organs of Respiration

**Published:** 1903-03

**Authors:** 


### Reviews of Books.

**Diseases of the Organs of Respiration.** By SAMUEL WEST, M.D. Vols. I. and II.: Pp. xii., 398; viii., 399–913. London: Charles Griffin & Company, Limited. 1902.

It is no light task that the author has undertaken, to place before the medical profession a new treatise on the organs of respiration, which does more than merely cover ground already so well occupied. But the progress that marks our knowledge of diseases of this region renders even recent treatises capable of improvement; and Dr. Samuel West has done so much to advance that knowledge that this new work is certain to be regarded as a valuable guide in practice. We congratulate the author not only on the matter, but also on the manner in which he has set it before the reader, for it is not given to every scientist to be so good a teacher of his subject.

We are pleased to note that Dr. West does not consider that all forms of membranous laryngitis are diphtherial, and he clearly states his conviction that a number of cases must be regarded as the result of other infective micro-organisms. In the laryngeal section there are some defects. Thus in discussing the pathology of congenital laryngeal stridor, the author, though relying on Sutherland and Lack's observations for the clinical appearances of the larynx in this affection, passes over their views as to the way in which the condition arises, and speaks of Thomson and Turner's experiments on the cadaver as conclusively demonstrating the causation. Again, the cadaveric position of the vocal cords is inaccurately stated to represent "the position of equilibrium or rest." The author, in discussing the pathology of abductor paralysis, and the early failure of the abductors, as compared with the adductors, makes absolutely no reference to "Semon's law," or to the researches of Semon and Horsley bearing on the question. Among the symptoms of malignant disease of the larynx, hoarseness is mentioned incidentally, but pain is stated to be a common symptom, "and being, in the absence of perichondritis, rare in other laryngeal affections (!), it becomes of some diagnostic importance." The section on laryngeal diseases is in several respects meagre, and taken as a whole cannot be regarded as worthy of a "special treatise" on this region, though a great advance on most text-books of medicine.

We wonder that Jenner's name finds no mention in connection with the expiratory theory in the production of emphysema, though so many others are mentioned as the expounders of various views.

The author does not uphold strongly the advantages of the sanatorium treatment of consumption; his praises of it are certainly faint. But we find little evidence of personal experience of sanatorium methods. We note with regret that no mention of the fact that open-air treatment was first introduced by an English physician named Bodington, although it is undoubtedly due to Germany that the method has been placed on a practical basis. We do not agree that "of Continental institutions the most highly developed at the present time is at Nordrach," or that "sanatorium treatment . . . will not cure the disease," or that "the sanatorium cure . . . is but a cheaper substitute for residence abroad."

As a treatise on diseases of the lungs and pleura, this work must take a very high place, and is worthy of the well-founded reputation of the author.

